# An assessment of implementation of Community-Oriented Primary Care in Kenyan family medicine postgraduate medical education programmes

**DOI:** 10.4102/phcfm.v8i1.1064

**Published:** 2016-12-02

**Authors:** Ian J. Nelligan, Jacob Shabani, Stephanie Taché, Gulnaz Mohamoud, Megan Mahoney

**Affiliations:** 1 Division of Primary Care and Population Health, Stanford University, United States; 2 Department of Family Medicine, Aga Khan University, Kenya; 3 Department of Family and Community Medicine, University of California, United States

## Abstract

**Background and objectives:**

Family medicine postgraduate programmes in Kenya are examining the benefits of Community-Oriented Primary Care (COPC) curriculum, as a method to train residents in population-based approaches to health care delivery. Whilst COPC is an established part of family medicine training in the United States, little is known about its application in Kenya. We sought to conduct a qualitative study to explore the development and implementation of COPC curriculum in the first two family medicine postgraduate programmes in Kenya.

**Method:**

Semi-structured interviews of COPC educators, practitioners, and academic stakeholders and focus groups of postgraduate students were conducted with COPC educators, practitioners and academic stakeholders in two family medicine postgraduate programmes in Kenya. Discussions were transcribed, inductively coded and thematically analysed.

**Results:**

Two focus groups with eight family medicine postgraduate students and interviews with five faculty members at two universities were conducted. Two broad themes emerged from the analysis: expected learning outcomes and important community-based enablers. Three learning outcomes were (1) making a community diagnosis, (2) understanding social determinants of health and (3) training in participatory research. Three community-based enablers for sustainability of COPC were (1) partnerships with community health workers, (2) community empowerment and engagement and (3) institutional financial support.

**Conclusions:**

Our findings illustrate the expected learning outcomes and important community-based enablers associated with the successful implementation of COPC projects in Kenya and will help to inform future curriculum development in Kenya.

## Introduction

When Moi University Faculty of Health Sciences launched the first family medicine postgraduate programme in Kenya in 2004–2005, training in population-based approaches to health care delivery was recognised as an essential component of the curriculum.^1^ Community-based education programmes such as Community-Oriented Primary Care (COPC) have been developed to provide real-world educational experiences in population health.^2,3,4,5,6,7^ Growing recognition of the value of the COPC process in family medicine training has recently prompted several postgraduate programmes in Kenya to include COPC in their curricula. Moi University led this innovation by implementing a COPC curriculum in 2011. Aga Khan University East Africa (AKU) established a family medicine postgraduate programme in Nairobi, which incorporates a multi-year longitudinal COPC project in 2012.

COPC is a systematic approach to community engagement based on principles derived from epidemiology, primary care, preventive medicine and health promotion with demonstrated positive health benefits for communities worldwide.^2,8^ The COPC model encourages public health practitioners, community leaders and health professionals to work collaboratively to identify and implement community-based health care interventions.^9,10,11^ As an integral part of many family medicine curricula, postgraduate students have completed COPC projects that have made lasting impacts in underserved communities and given postgraduate students an important skill set applicable to any health care setting.^6,12,13^

A qualitative assessment of stakeholders and current COPC practitioners in these two family medicine residencies in Kenya was conducted to explore experiences with community engagement, factors that contribute to programmatic success and current best practices at these institutions. The results will help to inform future development of COPC curriculum in Kenya. This project is part of a long-term academic partnership between the University of California San Francisco (UCSF) and AKU family medicine postgraduate programmes.

## Research methods and design

### Study design

We conducted a qualitative study based on focus group discussions (FGD) and key informant interviews with family medicine postgraduate students and faculty members at the two Kenyan family medicine residencies with COPC programmes at the time of the study.

### Setting

This study was conducted in the first year of the AKU family medicine residency programme at AKU Hospital in Nairobi, Kenya. The researchers included faculty from AKU, UCSF and a family medicine postgraduate student from UCSF with shared interests in medical education, COPC pedagogy and COPC implementation. The UCSF faculty shared experience with COPC implementation in family medicine training at UCSF since 2000 and learned from experienced collaborators from AKU. Interviews were conducted in Eldoret, Kangundo, Maragua and Nairobi, all in Kenya, by members of the research team in English.

### Study population and sampling strategy

We conducted two FGD, one with all current AKU residents^2^ in Nairobi and one with all Moi residents that were available to attend in Eldoret.^4^ Key informant interviews were scheduled with current AKU Family Medicine Faculty^9^ (M.M., J.S. and G.M. recused themselves because of participation in this study) and Moi faculty^9^ that were involved in the implementation of the Moi COPC curriculum. Because of time and scheduling constraints, postgraduate faculty members were interviewed individually, whereas postgraduate students participated in focus group interviews. Participants were invited via email and signed a written consent prior to participation.

### Data collection

Semi-structured interviews (lasting 30–60 minutes) and focus groups (lasting 90–120 minutes) were conducted by I.J.N., J.S. and G.M., exploring participants’ perspectives and best practices in COPC curriculum design and implementation in the Kenyan context between January and February 2013. The questions used in the interview guide are listed below:

- Have you participated in a project involving community engagement before?
- What did you learn from your COPC/community engagement experience, what challenges did you overcome?
- What does Community-Oriented Primary Care, or COPC for short, mean to you?
- Where did you first learn about COPC?
- What is an example of a COPC project you know about that has been successful?
- What are some of the challenges/barriers to COPC at your university? In Kenya?
- In which community are you most interested for a COPC project?
- What skills are most important for you [the residents] to learn through COPC?
- How do you [the residents] anticipate using COPC in your [their] careers?

### Data analysis

Recordings were transcribed, checked and corrected for any errors. The researchers then familiarised themselves with the data and developed a codebook. All data were coded and thematically interpreted independently by two researchers (M.M. and S.T.). Coding was reviewed for agreement, and common themes were synthesised by I.J.N. Discrepancies were resolved by discussion.

## Ethical considerations

The Aga Khan University Faculty of Health Sciences Research and Ethics Committee and University of California San Francisco Committee on Human Research approved the research protocol.

## Results

Two AKU faculty stakeholders in the Family Medicine Department, three AKU family medicine postgraduate students, five Moi University postgraduate students and three Moi University faculty members participated in the study.

Two broad themes emerged that are associated with a successful family medicine postgraduate COPC programme in Kenya: expected learning outcomes and community-based enablers of sustainability of COPC projects. Participants defined three learning outcomes of the COPC curriculum in Kenya. The first outcome focuses on how to make a community diagnosis,^14^ which ensures that trainees start the COPC process with a needs assessment and engagement of community members so the project addresses a priority of the community.

‘Community diagnosis means assessing the real health need of that particular group of people within that particular time in a particular geographical location, instead of making a decision of intervention without knowing what really ails the particular population in question.’ (Participant 3, Male, Faculty, Moi)

‘Before someone embarks on this project should know the health system of that community or area or region of the country where they are working. How things move? What is the responsibility of each individual, each post? Right? How to get joined into this community? Those are the basics skills that somebody should have.’ (Participant 2, Male, Faculty, AKU)

The second outcome emphasised the importance of understanding local environmental impacts on health outcomes and identified challenges in teaching these concepts. Through COPC projects, students learn about social determinants of health, cross-cultural communication and cultural sensitivity.

‘Communication skills are very critical…[*the students*] need to understand the culture, the religion, interpersonal communication, even literacy level of community.’ (Participant 1, Male, Faculty, Moi)

‘[*The residents*] say that [*COPC*] actually helps them understand what primary health care is all about.’ (Participant 2, Male, Faculty, AKU)

Finally, stakeholders discussed the value of training in participatory research, which includes community engagement, programme planning, implementation, evaluation, proposal writing and interpersonal communication skills.

‘I think it is those skills that you acquire, provide you a great opportunity to engage the community in terms of what is happening,… you will be able to engage the community much better and also be able to incur change in terms of behavior.’ (Participant 3, Female, Resident, AKU)

The second broad theme was the importance of three community enablers of COPC project sustainability. Participants highlighted partnering with community health workers (CHWs) as the first step to facilitate linkages to the community. There was consensus that COPC in Kenya must utilise CHWs to bridge health centres to the community.

‘[*Community health workers*] will be able to give you an indicator of the health status in the community.’ (Participant 2, Male, Resident, AKU)

‘When you involve [*community health workers*], they are actually the ones who would be able to guide you in actually being able to know the exact need of the community. I think they are in better position to know what the needs of the community are.’ (Participant 1, Female, Resident, AKU)

‘The community health workers are a summary of the big communities.’ (Participant 4, Male, Faculty, Moi)

Participants also described the value of community empowerment and engagement through local project ownership. A participant designed an education intervention for CHWs to address a problem observed at the district hospital level but the curriculum was never used because of lack of initial buy-in at the community level. He later revised the curriculum with community input and had improved the long-term success.

‘I think the essence behind a community engagement is to make sure that the community kind of takes responsibility of things.’ (Participant 1, Male, Resident, Moi)

‘[*Community engagement*] is the only way [*a COPC project*] will be able to sustain itself.’ (Participant 2, Male, Resident, AKU)

Finally, participants reported financial constraints, such as the cost of transport and payments expected by CHWs and community members, which can threaten the sustainability of COPC projects. The financial support from home institutions is essential for COPC project viability and success.

‘Community health workers are volunteers. So if you are calling them for a meeting, it would be important to provide them with a lunch and sometimes transport….’ (Participant 1 Male, Faculty, Moi)

‘It has proved to be quite an uphill task because of who is financing….’ (Participant 3, Female, Resident, AKU)

## Discussions

Our findings illustrate the expected learning outcomes and important community-based enablers of the successful implementation of COPC projects in Kenya and will help to inform future curriculum development. Participants agreed on the importance of learning key participatory research concepts and skills, such as community assessment and programme evaluation, and noted the value of cultural sensitivity, elements that are consistent with findings of COPC evaluations in the Western literature.^6,15^

The Kenyan government has deployed CHWs to improve health outcomes by bridging the gap between households and the health system. CHWs disseminate public health messaging and deliver basic health services at the community level. There was consensus that engaging CHWs improved the potential for project success by linking health centres to the community and increasing community ownership of the project. This is supported by the experience of the Community Based Education Research and Service (COBERS) programmes in Uganda where the lack of inclusive engagement with community stakeholders was found to limit programme success.^16^

Future studies should capture the impact of COPC projects on community health outcomes, clinical services and student career trajectory. Similar programmes in South Africa have seen improvements in clinical services, streamlined referral to district level hospitals, increased home visits and improved communication with patients.^17^ Longitudinal rural and underserved electives have been shown to successfully return students to practice in these areas; it would be valuable to monitor this effect among COPC programmes in Kenya.^18^ We also wonder about the potential for a universal COPC curriculum. Shared curricular components between the COPC curriculum in Kenya and established curricula in other African nations and the West would support the development of a universal open access online COPC curriculum. Future research could involve a comparative analysis of COPC curriculum in different cultural contexts to assess a universal competency-based COPC curriculum.

The study design could have resulted in overrepresentation of faculty members’ comments; however, we believe the themes that emerged were represented in both FGD and interviews, and would be informative for COPC development in Kenyan postgraduate training.

From this study, we characterise elements for successful implementation of COPC curriculum in Kenya to identify barriers and enablers unique to these programmes. The results of the study may help inform curriculum development at the three nascent family medicine postgraduate programmes that are currently under development in Kenya. Postgraduate training in COPC is valuable for building a pipeline of health professionals who are proficient in a participatory approach to community-based interventions that improve population health.
